# Drug–Drug Interactions Between Oral Anticoagulants and Calcineurin Inhibitors in Nephrotic Syndrome for Management of Thromboembolism

**DOI:** 10.3390/biomedicines14081666

**Published:** 2026-07-24

**Authors:** Toshinori Hirai, Kan Katayama

**Affiliations:** 1 Department of Pharmacy, Institute of Science Tokyo Hospital, 1-5-45 Yushima, Bunkyo-ku, Tokyo 113-8519, Japan; 2 Department of Cardiology and Nephrology, Mie University Graduate School of Medicine, Tsu 514-8507, Japan

**Keywords:** calcineurin inhibitor, tacrolimus, cyclosporine, voclosporin, anticoagulants, warfarin, direct oral anticoagulants, pharmacokinetics, drug-drug interaction, P-glycoprotein

## Abstract

Thromboembolism is a life-threatening complication of nephrotic syndrome, which is treated with oral anticoagulants (warfarin and direct oral anticoagulants [DOACs]) that possess a variety of pharmacokinetic characteristics. This narrative review focused on drug–drug interactions between oral anticoagulants and calcineurin inhibitors (CNIs) (e.g., tacrolimus, cyclosporine, and voclosporin) for the management of thromboembolism in nephrotic syndrome. While warfarin has less potential for interacting with CNIs, DOACs carry a risk of drug–drug interactions with CNIs owing to the inhibition of drug-metabolizing enzymes and transporters, including cytochrome P450 3A4 (CYP3A4) and P-glycoprotein. Specifically, a significant increase in DOAC exposure was observed when DOACs were co-administered with cyclosporine, a more potent P-glycoprotein inhibitor than tacrolimus. Limited evidence suggests that more pronounced drug interactions occur in specific cases: (1) apixaban and CNIs in patients with renal impairment, (2) edoxaban combined with cyclosporine in patients with renal impairment, and (3) a combination of rivaroxaban with dual inhibitors of P-glycoprotein and CYP3A4 (e.g., cyclosporine and fluconazole). From a pharmacological viewpoint, CNI-induced nephrotoxicity and hypertension may increase the risk of critical bleeding in patients receiving DOACs. In addition, the variation factors of pharmacokinetic parameters, such as renal function, pharmacogenomic profiles, and pathophysiological changes directly caused by nephrotic syndrome, may vary between patients, which could potentially augment the magnitude of drug–drug interactions between oral anticoagulants and CNIs. Further studies are required to understand the clinical significance of drug interactions in patients with nephrotic syndrome.

## 1. Introduction

Nephrotic syndrome, a major kidney disease, arises from various etiologies, including primary causes—such as minimal change disease, membranous nephropathy, and focal segmental glomerular sclerosis—and secondary causes—such as diabetic nephropathy and systemic lupus erythematosus [1]. Nephrotic syndrome is clinically characterized by edema, hypoalbuminemia, and heavy urinary protein exceeding 3.0–3.5 g per 24 h in adults [2].

Thromboembolism is a life-threatening complication of nephrotic syndrome [3]. During the first six months of follow-up, the annual cumulative incidence of venous and arterial thromboembolism was reported to be approximately 9.9% and 5.5%, respectively [4]. Traditionally, venous thrombosis has been regarded as the predominant thrombotic event underlying nephrotic syndrome [5]. However, recent epidemiological research has demonstrated that venous and arterial thrombotic risks are significantly increased in patients with nephrotic syndrome compared to the general population, with the magnitude of risk varying according to the underlying kidney disease [6]. The thromboembolic risk significantly differs between adults and children, with thromboembolism occurring in approximately 25% of adults compared to only 3% of pediatric patients [3].

Anticoagulant therapy (warfarin and direct oral anticoagulants [DOACs]) is a key intervention for preventing and treating thromboembolism in patients with nephrotic syndrome, despite the lack of definitive clinical evidence. However, oral anticoagulants potentially interact with calcineurin inhibitors (CNIs), which are frequently used as steroid-sparing agents to mitigate glucocorticoid-induced adverse effects, such as cardiovascular and metabolic disorders [7]. In addition, nephrotic syndrome itself may lead to pharmacokinetic changes through edema and hypoalbuminemia, thereby affecting drug distribution and clearance. Moreover, direct clinical evidence regarding drug–drug interactions between anticoagulants and CNIs in nephrotic syndrome remains limited, although several pharmacological considerations have been extrapolated from studies in healthy volunteers, transplant recipients, and non-nephrotic populations. Understanding the pharmacokinetic interactions between anticoagulants and CNIs is essential to optimize treatment efficacy and patient safety.

Existing reviews have addressed anticoagulation in nephrotic syndrome or drug–drug interactions involving DOACs independently. However, evidence regarding the combined impact of nephrotic syndrome-related pharmacokinetic alterations and calcineurin inhibitor co-administration on DOAC exposure has not been comprehensively synthesized.

This narrative review aims to summarize the current knowledge on drug–drug interactions between oral anticoagulants and CNIs in patients with nephrotic syndrome from the perspective of clinical pharmacology.

## 2. Pathophysiology and Management of Thromboembolism in Nephrotic Syndrome

The pathophysiology of thrombogenesis in nephrotic syndrome is characterized by a complex thrombophilic and hypercoagulable state [3].

Inflammatory and immune-mediated responses contribute to dysregulation of the coagulation cascade [8,9,10]. Nephrotic syndrome results in the urinary loss of negatively charged antithrombotic proteins such as antithrombin III and proteins C and S. Consequently, hepatic synthesis of several prothrombotic proteins is increased to compensate for protein loss, including factors V and VIII, von Willebrand factor, α_2_-plasmin inhibitor, plasminogen activator inhibitor 1, and fibrinogen. In addition, platelet hyperaggregation is frequently observed in nephrotic syndrome, which further contributes to the thromboembolic risk [11]. Renal vein thrombosis is partially explained by intravascular volume depletion and hemoconcentration, particularly in patients receiving diuretics [5]. Furthermore, patients with nephrotic syndrome who are carriers of two genetic variants (e.g., factor V) have been proposed to be at an additional risk of thromboembolism [12]. Consistent with pathophysiological mechanisms, a higher risk of thromboembolism is observed in patients harboring traditional risk factors, such as the male sex and older age, as well as laboratory abnormalities, including severe hypoalbuminemia, urinary protein, and elevated D-dimer [4,13,14,15]. Among the pathological subtypes, membranous nephropathy is associated with the highest thromboembolic risk, followed by focal segmental glomerulosclerosis [14].

Nevertheless, prophylactic anticoagulation remains unestablished [16]. Limited evidence suggests that thromboprophylaxis is preferable, considering the balance between thrombosis and bleeding, using HAS-BLED risk stratification scores not validated in patients with nephrotic syndrome [17]. The HAS-BLED score is estimated for bleeding risk with a maximum of 9 points based on seven items, including hypertension (1 point), abnormal renal/liver function (each 1 point), stroke (1 point), bleeding history (1 point), labile international normalized ratio (1 point), older age, defined as age ≥ 65 years (1 point), and drugs/alcohol concomitantly (each 1 point). A score of 0 indicates low risk, 1–2 indicates moderate risk, and ≥ 3 indicates high risk. This score supports the impact of drug interactions on bleeding risk during anticoagulation therapy. As patients with nephrotic syndrome have an increased risk of bleeding compared to the general population [6], close monitoring of bleeding manifestations is necessary.

A retrospective single-center analysis with a small sample size demonstrated a similar prophylactic effect between warfarin and DOACs, although a significantly lower risk of major bleeding was observed with DOACs than with warfarin (8.4% versus 0%, *p* = 0.045) [18]. Notably, patients receiving DOACs had a higher proportion of rituximab therapy and a shorter duration of anticoagulation therapy [18]. In contrast, a meta-analysis indicated that DOACs have a significantly higher risk of thrombosis and a lower risk of bleeding than standard care (warfarin or heparin) [19]. In terms of general management, oral anticoagulant therapy in patients with nephrotic syndrome is broadly similar to standard practice applied to patients without nephrotic syndrome.

## 3. Pharmacology and Pharmacokinetics

### 3.1. Overview

**Figure 1:**
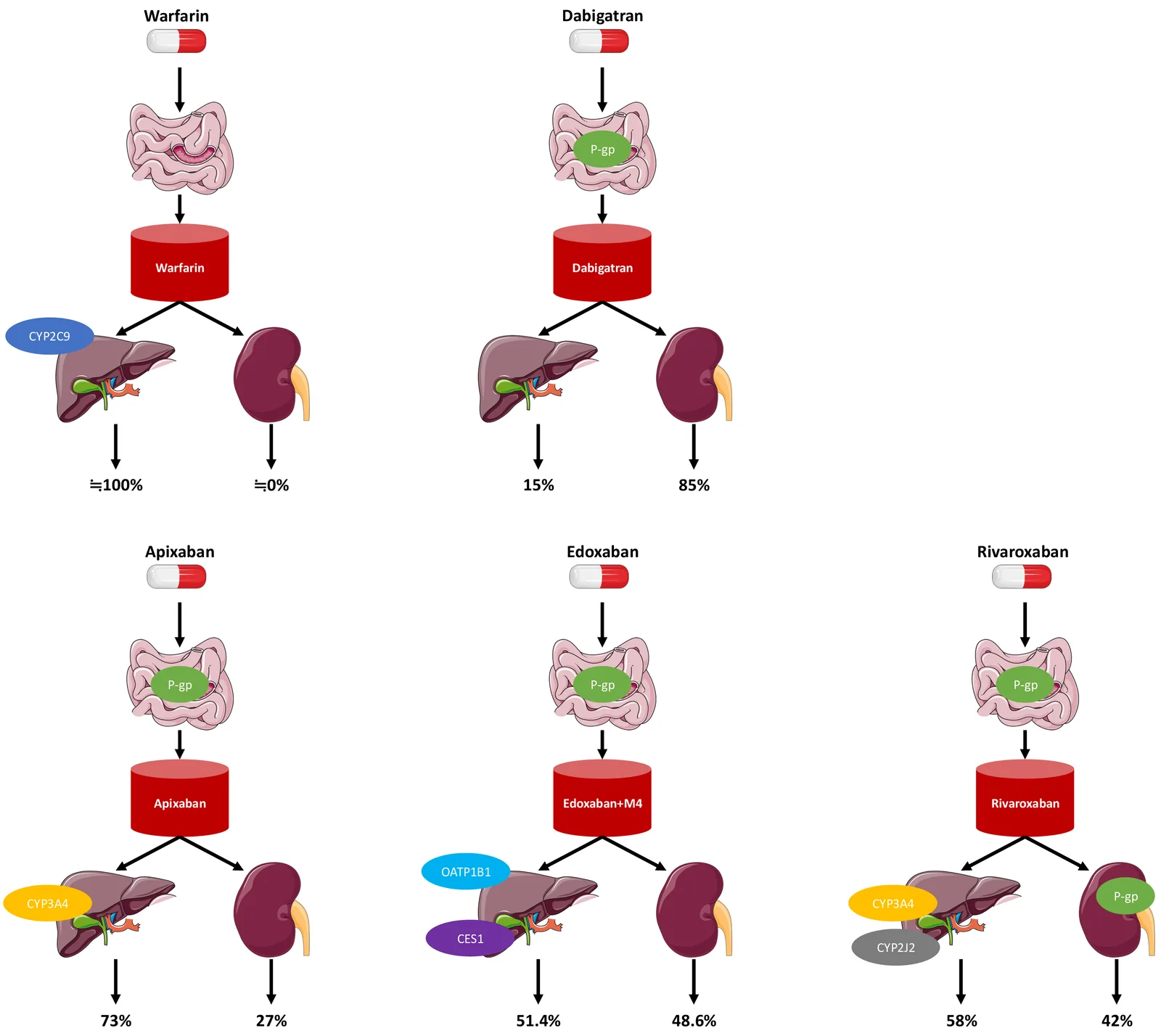
Pharmacokinetic pathway of warfarin and direct oral anticoagulants. Abbreviations: CYP, cytochrome P450; P-gp, P-glycoprotein; CES, carboxylesterase; OATP, organic anion transporting polypeptide. OATP1B1 uptakes M4 (an active metabolite of edoxaban).

Our review did not include discontinued DOACs, namely betrixaban. The detailed pharmacokinetics of the oral anticoagulants are presented in Table 1 and Figure 1 [20,21,22,23,24].

### 3.2. Warfarin

Warfarin is a racemic mixture of R and S enantiomers, and S-warfarin is roughly three times more potent than R-warfarin [25]. Warfarin inhibits vitamin K epoxide reductase complex subunit 1 (VKORC1), thereby impairing γ-carboxylation at the N-terminals of vitamin K-dependent coagulation factors such as prothrombin, preventing the formation of venous thrombosis [26]. After nearly 100% absorption of warfarin from the gut, the drug is bound to albumin in the blood and distributed in the extracellular fluid [24]. S-warfarin undergoes hepatic metabolism via cytochrome P450 (CYP) 2C9, whereas R-warfarin is a substrate for other CYP isozymes such as CYP2C19 and CYP3A4. A large inter-subject variability exists in the anticoagulant effect of warfarin due to pharmacokinetic and pharmacodynamic variability caused by CYP2C9 and VKORC1 polymorphisms as well as numerous factors, including age, body size, and concomitant medications [27,28,29].

### 3.3. DOACs

DOACs include a direct thrombin inhibitor (dabigatran) and factor Xa inhibitors (apixaban, edoxaban, and rivaroxaban). Several studies have supported the safety profile of DOACs compared with warfarin for the treatment of atrial fibrillation and thromboembolism [30]. However, DOACs do not show a net clinical benefit in patients with several conditions, including those with mechanical heart valves and antiphospholipid syndrome.

All DOACs are substrates of P-glycoprotein, an efflux transporter localized in the intestinal epithelium and proximal tubular cells, with varying degrees of contribution among agents [31]. The treatment doses for thromboembolism are summarized in Table 2. The required induction dose for apixaban was 10 mg twice daily for 7 days, and that for rivaroxaban was 15 mg twice daily for 21 days. Maintenance doses were fixed for all DOACs except edoxaban, which was administered at 60 mg once daily, or at a reduced dose of 30 mg once daily in patients with creatinine clearance (CLcr) of 30–50 mL/min, body weight < 60 kg, or concomitant use of P-glycoprotein inhibitors.

After oral administration, the inactive form of dabigatran etexilate is absorbed and hydrolyzed into an active moiety by esterase enzymes [36]. Dabigatran is equally distributed in extracellular and intracellular fluids and has a relatively low protein-binding ratio. This drug is predominantly eliminated in urine via glomerular filtration [37]. Because the clearance of dabigatran is largely equal to the glomerular filtration rate, tubular secretion via P-glycoprotein has a minimal contribution to the renal clearance of dabigatran.

Approximately 50% of apixaban is absorbed through the gastrointestinal tract [38]. Based on the volume of distribution, apixaban is predominantly distributed in the extracellular fluid. Approximately 90% of apixaban binds to proteins and is metabolized by O-demethylation, hydroxylation, and sulfation mediated by CYP3A4/5 in the liver, with minor contributions from other CYP isozymes such as CYP1A2, CYP2C8, CYP2C19, and CYP2J2 [39]. The circulating metabolites of apixaban do not exert an anticoagulant effect [40]. Apixaban is a substrate of P-glycoprotein and breast cancer resistance protein, as shown in an in vitro study [41].

The absolute bioavailability of edoxaban is approximately 60% [42]. Edoxaban is widely distributed throughout the body due to a large volume of distribution with a protein-binding ratio of approximately 50% [42]. Edoxaban is primarily metabolized by hydrolysis mediated by carboxylesterase 1 as well as conjugation and oxidation by CYP3A4/5 to a lesser extent. The active metabolite of edoxaban, M4, undergoes hepatic uptake via organic anion transporting polypeptide 1B1 (OATP1B1). M4 has a more potent half-maximal inhibitory concentration (IC_50_) than edoxaban (1.8 nM versus 3.0 nM); however, M4 does not elicit an anticoagulant effect owing to its low abundance and high protein binding [43].

Rivaroxaban is completely absorbed by the gut during fasting and is uniformly distributed throughout the body. Rivaroxaban undergoes hepatic metabolism through oxidation via CYP3A4 and CYP2J2 and hydrolysis via a non-CYP pathway [44]. Unmetabolized rivaroxaban is a major pharmacological compound in plasma [45]. Renal elimination—glomerular filtration and tubular secretion via P-glycoprotein and breast cancer resistance protein—partially contributes to the clearance of rivaroxaban. Indeed, a significant increase in rivaroxaban exposure was observed when healthy subjects concomitantly received fluconazole (a dual inhibitor of CYP3A4 and efflux transporters, P-glycoprotein and breast cancer resistance protein) [46].

## 4. Pharmacokinetic Changes in Nephrotic Syndrome

The well-stirred model describes drug clearance using the following equation [47]:

(1) $$CL=\frac{Q\times f_{u}\times {CL}_{int}}{Q+f_{u}\times {CL}_{int}}$$

where CL is the clearance, Q is the blood flow, f_u_ is the unbound fraction in the plasma, and CL_int_ is the intrinsic clearance. As described in “Pharmacology and Pharmacokinetics,” all anticoagulants are categorized as low extraction ratio drugs (Q >> f_u_ × CL_int_). The systemic exposure is approximated using the following equation:

(2) $$AUC=\frac{F\times Dose}{CL}=\frac{F\times Dose}{{f_{u}\times CL}_{int}}$$

where AUC is the area under the plasma drug concentration–time curve and F is the absolute bioavailability. Unbound clearance (CL_f_) is calculated by dividing CL by f_u_. The area under the unbound plasma drug concentration–time curve (AUC_f_) can be expressed as follows.

(3) $${AUC}_{f}=\frac{F\times Dose}{CL/f_{u}}=\frac{F\times Dose}{{CL}_{int}}$$

Alterations in bioavailability and clearance involving CYP and P-glycoprotein due to drug–drug interactions directly influence exposure to free drugs, which is clinically relevant in patients receiving a combination of anticoagulants and CNIs. Although hypoalbuminemia, a well-known abnormality in patients with nephrotic syndrome, theoretically leads to a significantly increased unbound fraction when f_u_ < 20%, hypoalbuminemia has limited influence on the unbound drug concentration at steady state for a drug with a low extraction ratio because the unbound concentration is primarily governed by CL_int_ rather than f_u_ as shown in Equation (3). A reduction in the total plasma drug concentration associated with hypoalbuminemia may not necessarily reflect reduced exposure to the free form of drugs.

Edema causes weight gain due to fluid retention, thereby expanding extracellular fluid. Accordingly, drugs with a relatively small volume of distribution (<30 L) may exhibit increased distribution in the extracellular fluid in the presence of edema, whereas drugs with a large volume of distribution do not [47]. As shown in Equation (3), the AUC_f_ at steady state is generally unchanged regardless of the change in volume of distribution. Consistent with this finding, a pharmacokinetic study of apixaban in patients with nephrotic syndrome revealed a decrease in AUC without a significant change in the AUC_f_ [48]. Therefore, edema is unlikely to substantially alter unbound exposure to anticoagulants, although changes in the volume of distribution may affect total plasma concentrations and half-lives. Urinary loss of albumin-binding drugs has also been hypothesized to potentially decrease drug exposure in nephrotic syndrome, although its clinical relevance remains uncertain.

Thus, the interpretation of total plasma drug concentrations without considering unbound exposure may lead to serious errors in anticoagulant dose adjustments in patients with severe hypoalbuminemia. In this review, we mainly focus on the bioavailability and clearance involving drug–drug interactions between oral anticoagulants and CNIs, including CYP enzymes and P-glycoprotein.

## 5. Drug–Drug Interactions Between Anticoagulants and CNIs

### 5.1. Overview

**Figure 2:**
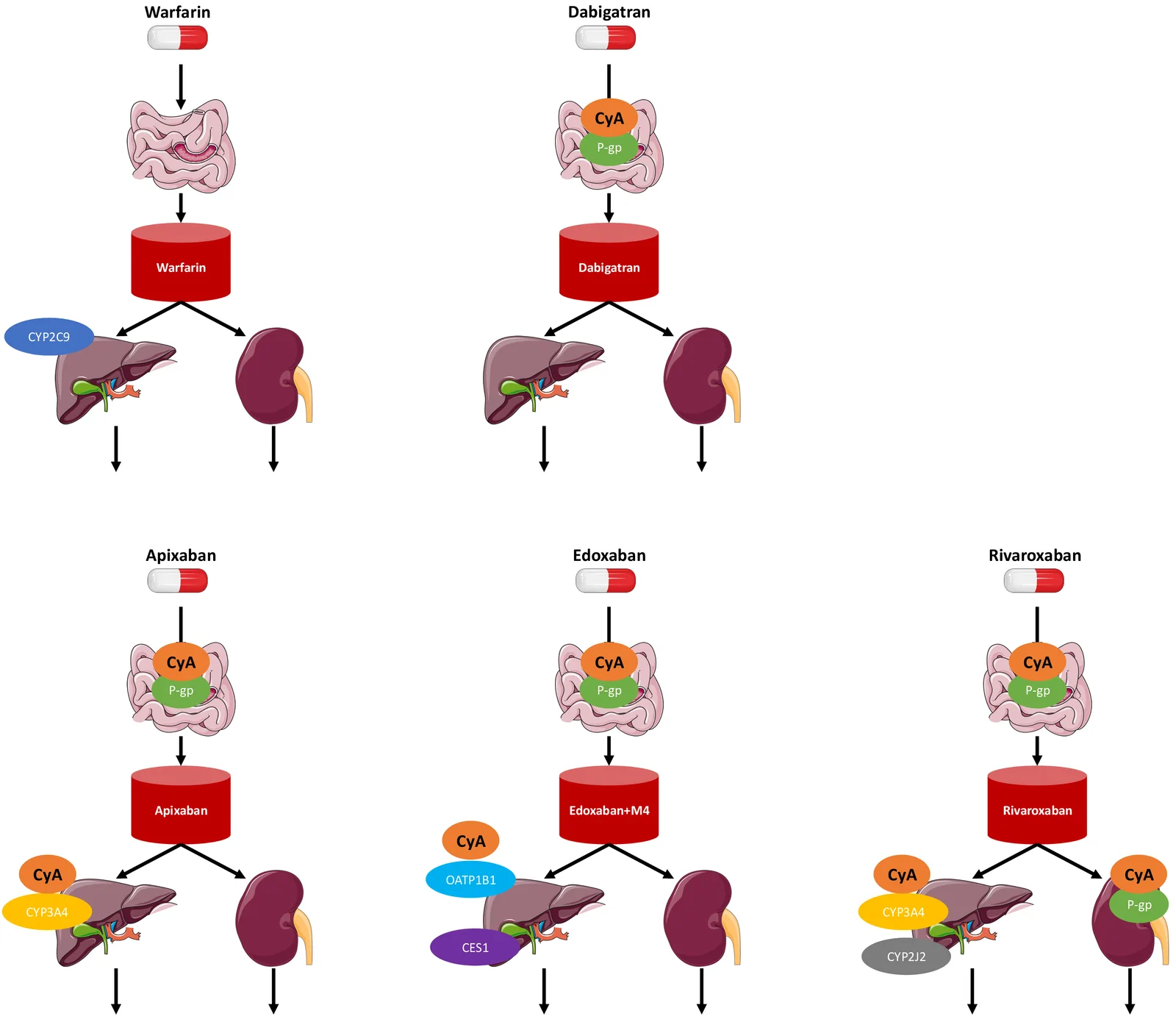
Drug–drug interaction between anticoagulants and calcineurin inhibitors. Abbreviations: CYP, cytochrome P450; P-gp, P-glycoprotein; CES, carboxylesterase; OATP, organic anion transporting polypeptide; CyA, cyclosporine. OATP1B1 uptakes M4 (an active metabolite of edoxaban).

Pharmacokinetic profiles vary among individual CNIs (Figure 2). The literature discussed in this section was identified through searches of PubMed, Web of Science, and Google. Following a narrative review style, no predefined systematic search strategy or fixed search terms were employed. Relevant publications were selected based on their relevance to the topic, with additional articles identified from the reference lists of key publications.

Conventional CNIs (tacrolimus and cyclosporine) are recommended for glucocorticoid-sparing therapy [49]. Tacrolimus is extensively metabolized via CYP3A4/5 and is a substrate of P-glycoprotein [50]. An in vitro study indicated that tacrolimus is a weak CYP3A4 inhibitor that is not clinically relevant [51]. In contrast, cyclosporine, a typical CYP3A4 substrate, moderately inhibits CYP3A4 and transporters such as P-glycoprotein, breast cancer resistance protein, and OATP1B1, which potentially increase the concentration of victim drugs [52]. Nonetheless, a large database analysis identified no association between major bleeding and co-prescription of DOACs and cyclosporine due to the rarity of the combination [53]. Nevertheless, the conversion of CNIs might alter the magnitude of the interaction with some DOACs (e.g., edoxaban and rivaroxaban). Given the limited information regarding drug–drug interactions, in vitro studies suggest that voclosporin, a novel CNI used to treat lupus nephritis, is a potential inhibitor of CYP3A4 and P-glycoprotein, with the possibility of clinically relevant drug–drug interactions [54,55]. Table 3 summarizes clinical decisions regarding anticoagulants according to the precipitating drug (mainly cyclosporine) and clinical consideration.

### 5.2. Warfarin

Evidence regarding drug–drug interactions between warfarin and CNIs is limited. One case report suggested the possibility of undesirable drug interactions, although a definitive conclusion could not be drawn [56]. Such an interaction is mechanistically unlikely, given that warfarin is primarily a CYP2C9 substrate, whereas cyclosporine acts as a CYP3A4 inhibitor. In an in vitro study, cyclosporine did not influence S-warfarin uptake into hepatocytes via organic anion transporter 2 [57]. Based on the findings, warfarin can be used to treat thromboembolism in patients with nephrotic syndrome to avoid drug interaction with CNIs. Given that sulfamethoxazole/trimethoprim is often co-prescribed to prevent pneumocystis pneumonia in patients with nephrotic syndrome, the concomitant use of sulfamethoxazole with warfarin should be considered with caution because of the inhibition of CYP2C9 [58,59].

### 5.3. Dabigatran

Transcellular transport studies have shown that cyclosporine and tacrolimus are potential inhibitors of the P-glycoprotein-mediated transport of dabigatran in the gastrointestinal tract [60]. Because CNIs potentially increase exposure to dabigatran owing to an elevation in bioavailability, systemic co-prescription of dabigatran and cyclosporine is contraindicated, and concomitant use with tacrolimus is not recommended in Europe [61]. However, no drug–drug interaction study has evaluated the impact of cyclosporine on dabigatran in clinical practice. As a reference, a common P-glycoprotein inhibitor, verapamil at 120 mg, administered 1 h before dabigatran administration at 150 mg, highlighted an increased AUC of 143%, despite no significant change in renal clearance [62]. This study emphasizes that the magnitude of drug interactions is minimized when dabigatran is administered 2 h before verapamil administration. Notably, the dosing interval between dabigatran and P-glycoprotein inhibitors should be considered because this drug interaction occurs in the gut. Furthermore, a tendency to increase the risk of major bleeding was observed in the dabigatran group compared to the apixaban group when co-administered with CNIs (mainly tacrolimus) after heart transplantation [63].

### 5.4. Apixaban

The co-administration of 100 mg cyclosporine or 5 mg tacrolimus changed the apixaban AUC when administered at 10 mg, with a geometric mean ratio of 119% and 77%, respectively [64]. These pharmacokinetic changes were within the margin of the clinical development process and did not require apixaban dose adjustment. Conversely, a significant increase in the AUC was found in lung and kidney transplant recipients compared to non-transplant recipients when apixaban was co-administered with either tacrolimus (4312 ng × h/mL versus 1511 ng × h/mL) or cyclosporine (5388 ng × h/mL versus 2356 ng × h/mL), although the precise mechanism remains unclear [65]. In this study, the CLcr for transplant recipients is numerically lower than that for non-transplant subjects (54 mL/min versus 141 mL/min), and trough concentrations were higher in transplant recipients for both tacrolimus (7.4 ng/mL versus 3.8 ng/mL) and cyclosporine (146.8 ng/mL versus 22.4 ng/mL). Decreased kidney function and drug interaction between CYP3A4 and P-glycoprotein in the hepatic presystemic process may contribute to increased exposure to apixaban. A transport assay suggested that breast cancer resistance protein is more sensitive to apixaban than P-glycoproteins [66]. When assessing the potential of drug interactions with apixaban, clinicians should prescribe both P-glycoprotein and breast cancer resistance protein inhibitors, as well as CYP3A4 inhibitors, with caution, particularly in special populations.

### 5.5. Edoxaban

Co-administration of cyclosporine at 500 mg once daily approximately doubled the edoxaban exposure, with similar effects observed with ketoconazole at 400 mg once daily, and erythromycin at 500 mg four times daily [67]. In this study, M4 exposure highlighted a 6.9-fold increase in the AUC in patients treated with edoxaban plus cyclosporine. This interaction occurred at the intestinal P-glycoprotein level for edoxaban, and OATP1B1 was expressed in hepatic cells for M4. A case series study implied that one out of 13 patients developed bleeding while receiving edoxaban 60 mg and oral cyclosporine 25 mg, without adherence to the dose reduction criteria described in the product information (body weight of 73.6 kg and CLcr of 59 mL/min) [68]. Because the gastrointestinal concentration is generally calculated based on the dose divided by 250 mL, the concentration of cyclosporine in the gut is markedly higher than that in the blood, even at a low dose of cyclosporine. Pharmacokinetic simulations predicted that the co-administration of cyclosporine in patients with severe renal impairment resulted in a 3.8-fold increase in edoxaban and a 22-fold increase in M4 exposure [69]. In addition, gene polymorphisms in P-glycoprotein and OATP1B1 did not influence exposure to edoxaban and M4 [70]. To avoid bleeding events associated with DOACs, it is essential to check the dosing criteria based on background factors such as body weight, kidney function (CLcr), and concomitant use of P-glycoprotein inhibitors.

### 5.6. Rivaroxaban

A marked increase in rivaroxaban AUC due to decreased tubular secretion via P-glycoprotein was observed in patients receiving a single 20 mg dose of rivaroxaban concomitantly with either cyclosporine alone or cyclosporine plus fluconazole at 400 mg once, with cyclosporine doses individualized to target trough concentrations of 70–100 ng/mL [71]. Based on this finding, the concomitant use of rivaroxaban with inhibitors of P-glycoprotein and CYP3A4 has been reported. In liver transplantation recipients, the trough concentration of rivaroxaban is clearly higher in the cyclosporine group than in the tacrolimus group (131.7 ng/mL versus 20.3 ng/mL, *p* = 0.014) under mean trough concentrations for cyclosporine of 69 ng/mL and tacrolimus of 3.4 ng/mL [72]. In contrast, cyclosporine exposure was slightly increased in patients receiving DOACs, particularly rivaroxaban, but not apixaban [73]. In a transporter analysis, tacrolimus in a clinically therapeutic range did not affect the P-glycoprotein-mediated efflux of rivaroxaban [74]. Rivaroxaban has no significant effect on the pharmacokinetics of tacrolimus in the clinical setting [75]. There were no effects on the pharmacokinetics of rivaroxaban and tacrolimus in either direction, without clinical events, such as bleeding and graft loss [76]. Clinical evidence is lacking regarding drug–drug interactions with rivaroxaban involving the CYP2J2 pathway. However, tacrolimus inhibits the metabolism of endogenous arachidonic acid (a probe of CYP2J2) in vitro, raising the possibility that CYP2J2 inhibition by tacrolimus might partially contribute to the risk of interaction of rivaroxaban [77].

### 5.7. Drug–Drug Interaction Involving Kidney Function and Blood Pressure

The risk of bleeding due to warfarin is higher as kidney function declines [78,79]. The population model predicted that a lower warfarin dose is required to attain the target prothrombin time–international normalized ratio (PT-INR) with a decline in kidney function due to vitamin K dysregulation in chronic kidney disease [80]. All DOACs are eliminated via the kidneys to a greater or lesser degree. CNIs predispose patients to the risk of nephrotoxicity, which is likely to occur in a concentration-dependent manner, particularly with supratherapeutic levels and long-term exposure [81]. The metabolism of CNIs is inhibited by the use of CYP3A4 inhibitors such as macrolides, azole antifungals, antiviral drugs, and calcium channel blockers [82,83,84,85], requiring dose adjustment of CNIs. Accordingly, personalizing the dosage of CNIs is a advisable approach for avoiding unexpected overexposure to DOACs. With a transient decline in the estimated glomerular filtration rate due to severe bilateral renal edema or acute nephrotoxicity associated with CNIs, a substantially increased risk of bleeding was observed with DOACs. When co-administered with CYP3A4 or P-glycoprotein inhibitors, renal impairment additively decreased DOAC elimination. Thus, selecting a DOAC with a lower contribution to renal clearance, such as apixaban, may offer a broader safety option for anticoagulation therapy in patients with acute nephrotic syndrome.

**Figure 3:**
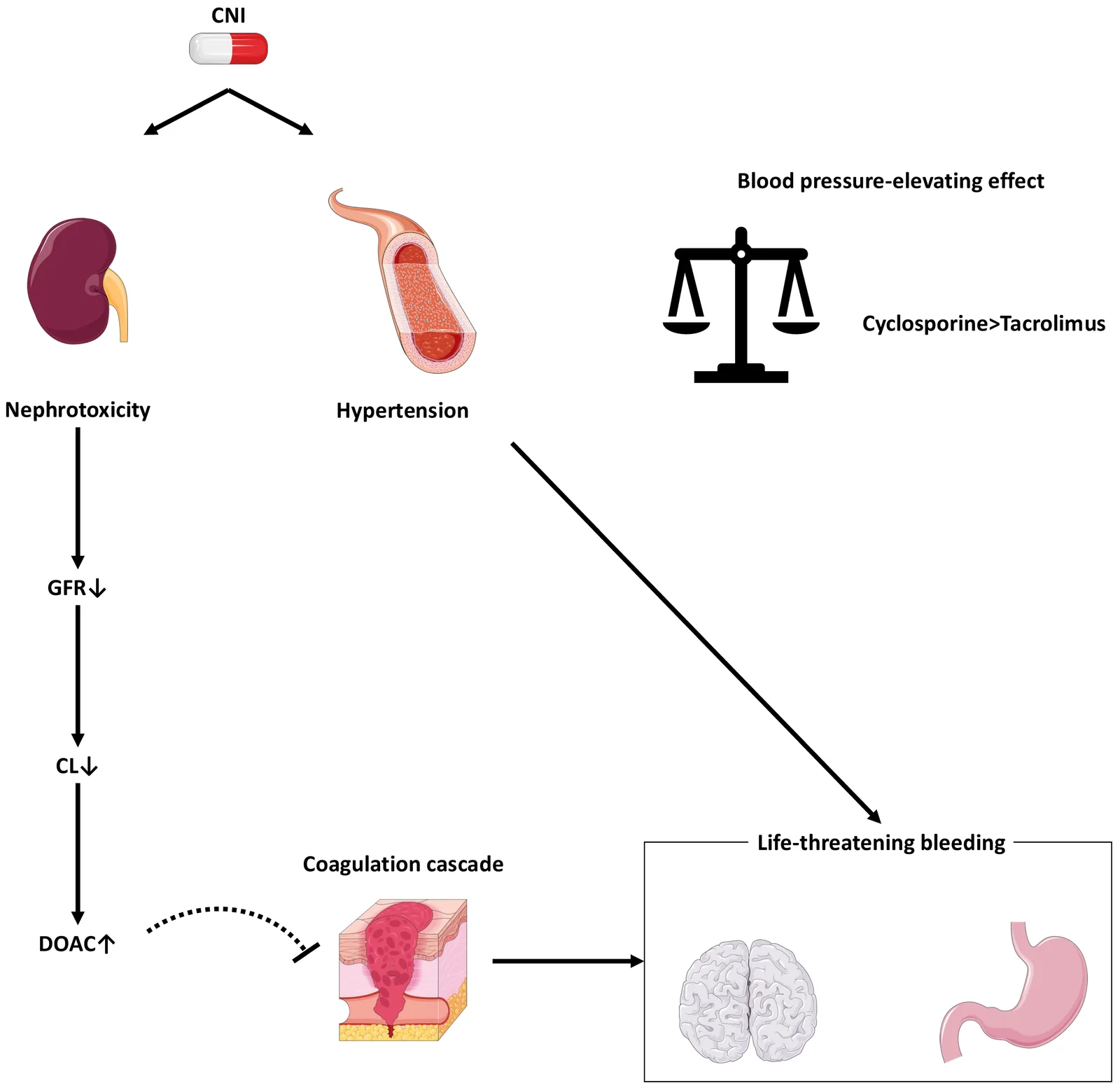
Pharmacological mechanism of increased risk of bleeding caused by drug–drug interaction between direct oral anticoagulants and calcineurin inhibitors. Abbreviations: CNI, calcineurin inhibitor; GFR, glomerular filtration rate; CL, clearance; DOAC, direct oral anticoagulants.

Similarly, hypertension is regarded as a common adverse effect of CNIs through the activation of the renin–angiotensin system, sympathetic nervous system, and imbalance in vasoconstriction and vasodilation (Figure 3) [86].

The risk of hypertension in transplant recipients is higher with cyclosporine than with tacrolimus [87,88]. In a basic experiment in rats, tacrolimus temporarily increased blood pressure, whereas voclosporin consistently elevated it [89]. Hypertension is an independent risk factor for lethal bleeding in patients undergoing anticoagulant therapy [90,91]. Thus, blood pressure monitoring plays an important role in the management of anticoagulant therapy combined with CNIs. Calcium channel blockers alleviate CNI-associated hypertension, especially cyclosporine, although certain calcium channel blockers intervene in the hepatic metabolism of CNIs via CYP3A4 [92]. Antihypertensive agents are valuable tools for controlling both blood pressure and the risk of bleeding events involving anticoagulants.

These pharmacodynamic interactions between anticoagulants and CNIs are similar in patients treated with a combination therapy of DOACs and nonsteroidal anti-inflammatory drugs, which potentially increases the risk of intracranial and gastrointestinal bleedings [93]. Clinical pharmacists should perform elaborate prescription checks with close follow-up of kidney function and blood pressure in collaboration with nephrologists.

## 6. Clinical Implications and Future Perspectives

Currently, guidance for the co-administration of CNIs and anticoagulants is extrapolated from evidence regarding drug–drug interaction studies in healthy volunteers and non-interventional studies in transplant recipients. Therefore, these findings should be interpreted cautiously when applied to patients with nephrotic syndrome. Disease-specific factors, including hypoalbuminemia, proteinuria, altered renal function, and concomitant medications, may influence both drug disposition and clinical outcomes.

Several randomized controlled trials evaluating apixaban and rivaroxaban have been planned to exclude patients receiving CYP3A4 inhibitors [33,35,94]. There is a critical shortage of large-scale prospective clinical trials or robust real-world evidence evaluating the efficacy and safety of DOACs, specifically within the nephrotic syndrome population. Future registry studies are urgently needed to clarify the clinical outcomes of patients receiving concurrent anticoagulant therapy and CNIs, including major bleeding events and thromboembolic recurrence.

Given the narrow therapeutic index of CNIs and the varying pharmacokinetic profiles of oral anticoagulants, a tailored approach is essential when co-administering these agents to patients with nephrotic syndrome. Cyclosporine, a potent inhibitor of both CYP3A4 and transporters (P-glycoprotein and breast cancer resistance protein), significantly increases the plasma concentrations of most DOACs. Therefore, when cyclosporine is indispensable, choosing an anticoagulant with lower P-glycoprotein dependency or considering preemptive dose reduction is warranted. Tacrolimus exhibits a minimal inhibitory effect on P-glycoprotein, making it a potentially safer alternative; however, strict therapeutic drug monitoring of tacrolimus remains mandatory.

Beyond the concept of drug–drug interactions, individual genetic variations in drug-metabolizing enzymes and transporters can further amplify the complexity of concomitant CNIs and anticoagulant therapies, leading to drug–drug–gene interactions [95]. For instance, genetic polymorphisms in ABCB1, which encodes P-glycoprotein, have been shown to modulate the transport efficacy of both cyclosporine and DOACs (e.g., rivaroxaban). Patients with specific ABCB1 variants such as the C3435T polymorphism may have altered P-glycoprotein expression in the intestine [96]. When a patient is exposed to cyclosporine, a potent P-glycoprotein inhibitor, the subsequent magnitude of interaction with DOACs can be unpredictable and highly variable.

Clinical management of drug–drug interactions is exceptionally challenging during the induction phase of nephrotic syndrome, which is characterized by severe edema and hypoalbuminemia. Because both CNIs and certain anticoagulants such as warfarin and rivaroxaban are highly protein-bounding, a drastic decline in serum albumin leads to an increase in the unbound fraction, but not the free concentration, of these drugs, as discussed in “Pharmacokinetic Changes in Nephrotic Syndrome”. Standard therapeutic drug monitoring, which measures total drug concentrations, may underestimate the actual pharmacological and toxicological activity owing to the free concentration of drugs. Ideally, a methodology for measuring free concentrations of anticoagulants is required to overcome these limitations. Clinicians should not only rely on measured trough levels of CNIs but also implement intensified monitoring of coagulation parameters, such as PT-INR for warfarin and clinical signs of bleeding or recurrent thrombosis. Although pharmacokinetic studies consistently demonstrate increased drug exposure with certain CNI combinations, clinical outcome data remain limited. While an increased exposure to anticoagulants may theoretically increase the risk of bleeding, particularly in patients with impaired renal function, robust evidence linking pharmacokinetic changes to clinical outcomes such as major bleeding or recurrent thromboembolism is lacking.

Consideration of the genetic background and pathophysiological changes in the nephrotic syndrome is crucial for comprehensive risk assessment in patients receiving DOACs. Nephrotic syndrome is characterized by a hypercoagulable state. However, it remains unclear whether the increased exposure to DOACs caused by drug interaction with CNI adequately offsets the underlying thrombotic risk without increasing bleeding complications. Currently, there is insufficient evidence to define this balance. Clinical studies are warranted to determine the optimal anticoagulation strategy.

## 7. Conclusions

The management of thromboembolism in patients with nephrotic syndrome requires a comprehensive understanding of the complex drug–drug interactions between oral anticoagulants and CNIs, which are further complicated by the pathophysiological changes associated with nephrotic syndrome. A multidisciplinary approach integrating clinical pharmacology, therapeutic drug monitoring, and individualized risk assessment is paramount for navigating these challenges and ensuring optimal outcomes in these patients.

## Figures and Tables

**Table 1:** Pharmacokinetic parameters of warfarin and direct oral anticoagulants.

	F, %	Ae, %	f_b_, %	Vd, L	CL, mL/min
Warfarin	93–94		97–99.5	7.6–13.9	1.6–8
Dabigatran	3–7	85	35	60–70	108–110
Apixaban	50	27	87	21	55
Edoxaban	61.8	48.6	40–58.9	107	363.3
Rivaroxaban	112 *	42	92–95	50	166.7

Abbreviations: F, absolute bioavailability; Ae, urinary excretion ratio; f_b_, protein binding ratio; Vd, volume of distribution; CL, clearance. *: 5 mg with fasting (unpublished data).

**Table 2:** Dosing schedule of direct oral anticoagulants for thromboembolism.

	Induction	Maintenance
Dabigatran [32]	-	150 mg twice daily
Apixaban [33]	10 mg twice daily for 7 days	5 mg twice daily
Edoxaban [34]	-	60 mg or 30 mg daily *
Rivaroxaban [35]	15 mg twice daily for 21 days	20 mg daily

*: For edoxaban, dose reduction is recommended if patients have creatinine clearance of 30–50 mL/min, body weight < 60 kg, or concomitant use of P-glycoprotein inhibitors.

**Table 3:** Guidance for combination of anticoagulants and cyclosporine.

	Target Molecule	Clinical Consideration
Warfarin	CYP2C9	No clinically relevant interactions
Dabigatran	P-gp	Contraindicated with cyclosporine
Apixaban	CYP3A4, P-gp, and BCRP	Renal impairment
Edoxaban	P-gp and OATP1B1 *	Renal impairment
Rivaroxaban	CYP3A4 and P-gp	Dual CYP3A4/P-gp inhibition

Abbreviations: CYP, cytochrome P450; P-gp, P-glycoprotein; BCRP, breast cancer resistance protein; OATP, organic anion transporting polypeptide. *: OATP1B1 uptakes M4 (an active metabolite of edoxaban).

## Data Availability

No new data were created or analyzed in this study. Data sharing is not applicable to this article.

## Abbreviations

The following abbreviations are used in this manuscript: DOAC, direct oral anticoagulants; CNI, calcineurin inhibitor; VKORC1, vitamin K epoxide reductase complex subunit 1; CYP, cytochrome P450; OATP1B1, organic anion transporting polypeptide 1B1; IC_50_, half-maximal inhibitory concentration; CL, clearance; Q, blood flow; fu, unbound fraction in plasma; CL_int_, intrinsic clearance; AUC, area under the plasma drug concentration–time curve; F, absolute bioavailability; CL_f_, unbound clearance; AUC_f_, area under the unbound plasma drug concentration–time curve.
